# Effects of global changes on the climatic niche of the tick *Ixodes ricinus* inferred by species distribution modelling

**DOI:** 10.1186/1756-3305-6-271

**Published:** 2013-09-19

**Authors:** Daniele Porretta, Valentina Mastrantonio, Sara Amendolia, Stefano Gaiarsa, Sara Epis, Claudio Genchi, Claudio Bandi, Domenico Otranto, Sandra Urbanelli

**Affiliations:** 1 Department of Environmental Biology, University of Rome “La Sapienza”, Via dei Sardi 70, 00185 Rome, Italy; 2 Department of Veterinary Science and Public Health, University of Milan, Milan, Italy; 3 School of Bioscience and Biotechnology, University of Camerino, Camerino, Italy; 4 Department of Veterinary Medicine, University of Bari, Bari, Italy

**Keywords:** Climatic changes, Future climatic niche, Species distribution modeling, Tick, Vector-borne disease, Lyme disease, Babesiosis, Anaplasmosis, Erlichiosis and tick-borne encephalitis

## Abstract

**Background:**

Global climate change can seriously impact on the epidemiological dynamics of vector-borne diseases. In this study we investigated how future climatic changes could affect the climatic niche of *Ixodes ricinus* (Acari, Ixodida), among the most important vectors of pathogens of medical and veterinary concern in Europe.

**Methods:**

Species Distribution Modelling (SDM) was used to reconstruct the climatic niche of *I. ricinus*, and to project it into the future conditions for 2050 and 2080, under two scenarios: a continuous human demographic growth and a severe increase of gas emissions (scenario A2), and a scenario that proposes lower human demographic growth than A2, and a more sustainable gas emissions (scenario B2). Models were reconstructed using the algorithm of “maximum entropy”, as implemented in the software Maxent 3.3.3e; 4,544 occurrence points and 15 bioclimatic variables were used.

**Results:**

In both scenarios an increase of climatic niche of about two times greater than the current area was predicted as well as a higher climatic suitability under the scenario B2 than A2. Such an increase occurred both in a latitudinal and longitudinal way, including northern Eurasian regions (e.g. Sweden and Russia), that were previously unsuitable for the species.

**Conclusions:**

Our models are congruent with the predictions of range expansion already observed in *I. ricinus* at a regional scale and provide a qualitative and quantitative assessment of the future climatically suitable areas for *I. ricinus* at a continental scale. Although the use of SDM at a higher resolution should be integrated by a more refined analysis of further abiotic and biotic data, the results presented here suggest that under future climatic scenarios most of the current distribution area of *I. ricinus* could remain suitable and significantly increase at a continental geographic scale. Therefore disease outbreaks of pathogens transmitted by this tick species could emerge in previous non-endemic geographic areas. Further studies will implement and refine present data toward a better understanding of the risk represented by *I. ricinus* to human health.

## Background

Global climate change can seriously impact on the epidemiological dynamics of vector-borne diseases [1-5]. However, because of the influence of several biotic and abiotic factors on hosts, arthropods and pathogens they vector, future spatial and temporal distribution of vector-borne diseases is still difficult to predict [4,6]. Under the above circumstances, predicting how climatic changes will affect geographic distribution of the vector is an obligate step. Indeed, vectors play a key role in infectious disease areas where they may transmit pathogens to a variety of animal hosts, often representing the bridge between zoonotic reservoirs and humans [3,7,8]. Likewise, since pathogens may disperse through arthropods into previously non-endemic areas, climate-induced changes in vector distribution ultimately affect the epidemiology of vector-borne diseases [3,9]. In addition, a distributional shift of vectors may also lead to spatial overlap of different vector species [10], thus changing the impact of pathogen transmission, interfering with their epidemic cycles [4] or as a consequence of vector interspecific competition [11-13].

In the last few years, Species Distribution Modelling (SDM) has greatly contributed to understanding the effect of climatic changes on vector distribution, combining known occurrence points of a species with a set of climatic variables [14,15]. Indeed, such an approach allows developing the potential geographic distribution under current and future climatic conditions [16-19]. SDM has been used in several studies on arthropod vectors [20-25], allowing to infer future range expansion, such as in the case of sand fly species *Lutzomyia anthophora* and *L. diabolica* in North America [22], or a potential distributional shift and/or a reduction of geographic range, as suggested for the malaria vectors in Africa [21].

In this study we investigated how future climatic changes could affect the climatic niche of the tick *Ixodes ricinus* (Acari, Ixodidae). This species is regarded as the most important vector occurring in Eurasian regions since it is a multi-competent vector of bacteria (i.e. *Borrelia burgdorferi* s.l., *Babesia* spp., *Anaplasma* and *Erlichia* spp.) and viruses (i.e. *Flavivirus* spp.), to humans and animals [26-30]. The above tick species is regarded as a major vector responsible for many zoonotic diseases, such as Lyme disease, babesiosis, anaplasmosis, erlichiosis and Tick-Borne Encephalitis (TBE) [31]. *Ixodes ricinus* has been recorded in Europe, Russia, up to the Caspian Sea on the east, and North Africa [32]. Along the western boundary of Russia and the neighboring countries, its range overlaps with the range of *I. persulcatus* tick, distributed in Eastern Europe and across Asia [30,33,34]. Recently, a latitudinal and altitudinal shift has been reported in *I. ricinus* distribution in European regions, and temperature rise was suggested to be among the factors responsible for this phenomenon [35-40]. In spite of the epidemiological implications that vector distribution changes might have on vectored pathogens, no studies have investigated the impact of future climatic changes on the geographic distribution of this tick species at a continental geographic scale.

By using SDM we aimed to reconstruct the climatic niche of *I. ricinus* and projected it into the future conditions for 2050 and 2080, under two possible scenarios: i) a continuous human demographic growth and a severe increase of gas emissions (A2 scenario), ii) a lower human demographic growth than A2, and a more sustainable gas emissions (scenario B2) [41].

## Methods

**Figure 1:**
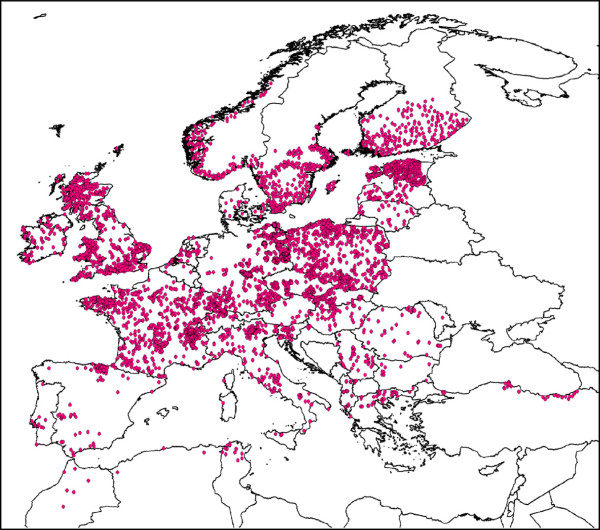
**Occurrence points.** Map shows the 4,544 records of *I. ricinus* used to develop the Species Distribution Modelling.

To reconstruct the current and future climatic niche of *I. ricinus*, the algorithm of “maximum entropy” has been used, as implemented in the software Maxent 3.3.3e. This technique, using presence-only points in conjunction with environmental variables, estimates the potential distribution of the species finding the probability distribution that approximates the distribution of maximum entropy [42]. This approach is largely used to reconstruct SDM because its performances are highly competitive with the other modeling methods. Indeed it showed the better calibration when compared to 16 other algorithms, including several traditional tools using presence-absence data, such as general linear models (GLM) and general additive models (GAM) [18,43-46]. To model the current and future climatic niche of *I. ricinus*, we used 4,544 occurrence points (Figure 1) obtained from the digital dataset available at https://sites.google.com/site/palticks/home/download[47]. The dataset includes occurrence points obtained by a systematic search of the published, peer-reviewed literature since, approximately, 1970 to 2010; records of ticks available in some curated collections were also included. It covers most of the *I. ricinus* range and does not include records considered as potentially incorrect, based on the known distribution of *I. ricinus*[47]. Environmental data were downloaded from WorldClim database (http://www.worldclim.org) with a resolution of 2.5 arc-minutes (~ 5 km). Nineteen bioclimatic variables derived from monthly temperature and rainfall values were downloaded, that represent annual trends, seasonality and extreme or limiting environmental factors. The runs have been made using 75% of the occurrence points to construct the model (training data) and the remaining 25% to test it. The default parameters of Maxent have been used with the exception of the Regularization parameter *β.* This parameter acts as a multiplier for the default values and it regulates the smoothness and regularity of the model [44,48]. Recently, Warren & Seifert [48] have highlighted the importance to test different values of this parameter to improve the Maxent’s performance. We developed ten models using different values of *β* (1, 3, 5, 7, 9, 11, 13, 15, 17 and 19) and then we chose the model that outperformed all the others using the sample sized corrected Akaike Information Criterion (AIC_c_) score, as implemented in the software ENMTools. The model built with the value of *β* equal to 1 gained the lowest score for the AIC_c_, thus outperforming all the remaining nine models tested, therefore it has been used to reconstruct the final models. To evaluate the accuracy of the developed models we used the area under the curve (AUC) of the receiver operating characteristic (ROC) as suggested by Peterson *et al.*[49]. Explorative analyses have been done using all 19 variables, then the environmental variables outside the range present in the training data have been excluded. The final models have been reconstructed using 15 variables (Additional file 1). Ten replicates were run using the cross-validation form of replication. This approach randomly split the data into equal-size groups (“folds”) and creates models leaving out each fold in turn and using them for evaluation [50]. The model developed for the present-day conditions has been projected onto the future climatic conditions. Two scenarios proposed by the *Special Report of Emission Scenarios* (SRES) of the Inter-governmental Panel on Climate Change (IPCC) [51], have been considered, namely: scenario A2 (that proposes a continuous human demographic growth and a severe increase of gas emissions) and scenario B2 (that proposes lower human demographic growth than A2, and a more sustainable gas emissions) [17,41]. Potential climatic niche under these scenarios has been predicted for 2050 and 2080 using the *Canadian Center for Climate Modeling and Analysis* CCCAM-CGCM3.1-T47 model [52].

All SDM predictions were visualized in Quantum-GIS 1.8.0 (http://download.qgis.org). To estimate the future increase of the niche area respect to current model, presence/absence maps using the minimum training presence threshold were constructed [53]. Then future increase of the niche area respect to current model was calculated using the software ImageJ (http://rsb.info.nih.gov/ij/).

## Results

**Figure 2:**
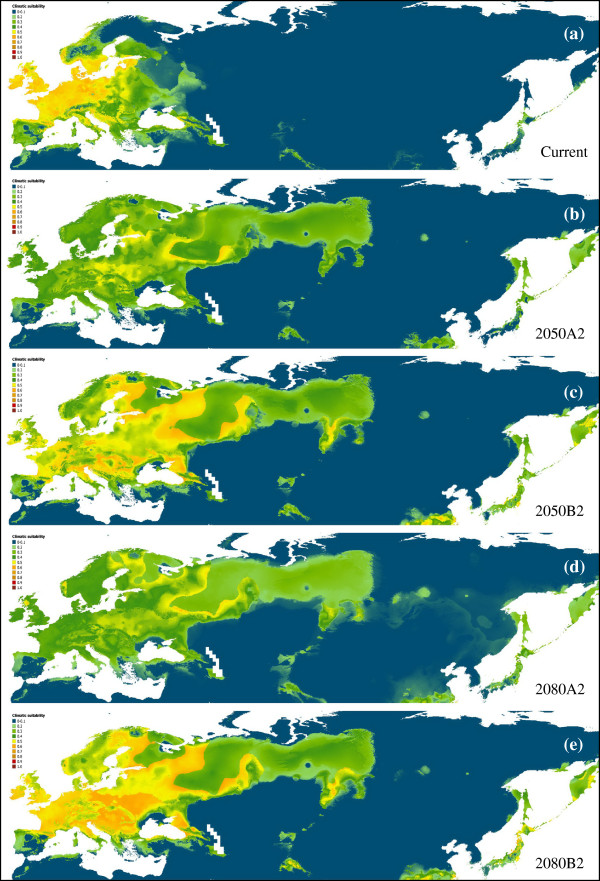
**Geographic distribution predicted by species distribution modelling for *Ixodes ricinus*. (a)** Current conditions; **(b**-**c)** 2050, scenario A2 and B2, respectively; **(d**-**e)** 2080, scenario A2 and B2, respectively.

The averaged climatic niche of the species for current conditions is shown in Figure 2a. The averaged value of AUC for this model was 0.860 (± 0.004), indicating an optimal performance of the models. Among the 15 variables used BIO6 (Min Temperature in the coldest period) and BIO17 (Precipitation of the driest quarter) give the highest percent contribution to construct the model (Additional file 1), according to the biology of *I. ricinus*, that lives in biotopes that offer moderate temperatures and high relative humidity [54]. The climatically suitable area predicted by our model under current conditions encompasses the known geographic distribution of *I. ricinus* and that previously inferred for this species using both climatic features and vegetation index [55], supporting the validity of our reconstruction.

In Figure 2b-e the models predicted under future climatic conditions are shown. In both scenarios an increase of a climatically suitable area of about two times greater than the current area was predicted (Additional file 2). Such an increase occurred both in a latitudinal and longitudinal way, including northern Eurasian regions (e.g. Sweden and Russia), that were previously unsuitable for the species. Notably, climatically suitable conditions appeared also in the easternmost Asian region, such as Central China, South Korea, Japan and Kamchatka Peninsula. While the two scenarios showed no difference about the extension of the predicted suitable area, they instead showed some difference in its climatic suitability. Indeed, under the scenario B2 most of the predicted area showed a higher climatic suitability than A2 in both 2050 and 2080. Furthermore, under the scenario B2 an increase of climatic suitability for *I. ricinus* has been predicted between 2050 and 2080 in several areas (i.e. Ireland and Eastern Europe) (Figure 2c,e).

## Discussion

The increase in temperature, which has been predicted to occur in the future years [41], could drastically affect the ecology and geographic distribution of *I. ricinus* in Eurasian regions. Climatic changes have been shown to affect seasonal activity and feeding behavior of *I. ricinus*, at different life stages [3,32,40]. Furthermore, because of the *I. ricnus* vulnerability to drought [54], global warming may affect negatively the species in southernmost part of its range of distribution. On the other hand, higher temperature could lead to milder winter and extended spring and autumn seasons than those actually characterizing northern regions, making these areas climatically suitable for the species. Evidence that this latter phenomenon is in progress in Europe has been already recorded for *I. ricinus*, in which an expansion of the northern distribution limits in Sweden and Norway has been reported since the 1980s [35,38-40,56]. Likewise, a shift of the limit to higher altitudes northward in Czech Republic and Switzerland respectively, has been shown [36,37]*.* Our results by using SDM are congruent with the predictions of range expansion and provide a qualitative and quantitative assessment of the future areas climatically suitable for *I. ricinus* at a continental scale.

While interpreting inferred expansion of climatic niche in the context of possible changes in geographic distribution of *I. ricinus*[15-18] the following two issues should be considered. First, the climatic features are only one of the ecological features that affect the geographic distribution of tick species [57,58]. However, *I. ricinus*, as all other ixodid ticks, spend most of their life off the host, so that climate is an essential determinant of their occurrence [59,60]. At a smaller geographic scale, other abiotic factors should be considered to assess the effective occurrence of *I. ricinus*, such as landscape physical features, or landscape use [15] and biotic factors (competition, hosts abundance etc.) [15,61,62]. The integration of these factors could show discontinuity in areas that our model predicted as large continuous areas of climate suitability. With respect to biotic factors, the hosts are key components for the ecological niche of ticks [7,63,64]. *Ixodes ricinus* is able to exploit a large variety of terrestrial vertebrates [7,63-65], so that host occurrence should not be a limiting factor to their persistence under climatic change scenarios [66]. Its wide ecological plasticity with respect to host choice, for example, was a key factor in allowing *I. ricinus* to survive during the last glacial phases without significant range reduction across the European continent [67].

Secondly, it should be considered that in order to have an effective range expansion, the new climatically suitable areas should be reached by the species. Host movements largely determine tick dispersal during infestation [65,68-72]. Among the several hosts exploited by *I. ricinus,* some of them, such as birds and cervids, are characterized by high dispersal ability. Host mediated dispersal of ticks also across long distances and more distant geographic areas have been demonstrated in several studies both for *I. ricinus*[72-74] and for other tick species [40,67,75-77]. Following the above considerations, although the use of SDM models at a higher resolution should be integrated by a more refined analysis of further abiotic and biotic data, the general significance of results presented here suggest that under future climatic scenarios, most of the current distribution area of *I. ricinus* could remain suitable and significantly increase at a continental geographic scale.

Our results suggest also that the more favourable climatic conditions for *I. ricinus* will occur under the scenario B2 (i.e., lower human demographic growth, and a more sustainable gas emissions than A2). Interestingly, reduced demographic growth and gas emissions are objectives of several international policies [78]. In this context it is further advisable to investigate the factors that could affect the epidemiological dynamics in which this tick is involved. For example, future inferred expansion eastward of the climatic niche of *I. ricinus* could increase the overlapping areas with the Eurasian tick *I. persulcatus*[79], increasing or decreasing pathogen transmission due to vector interspecific competition [11-13], or to interferences among pathogen epidemic cycles [4]. This may be the case for *Borrelia* spp. in which a relationship has been established between bacterial complexes and tick species. Indeed, while the Eurasian type of *B. garinii* and *B. afzelii* are carried by both *I. ricinus* and *I. persulcatus* in North Asian regions, *B. burgdorferi* s.s. seems to be vectored exclusively by *I. ricinus*, and the Asian type of *B. garinii* by *I. persulcatus*[80]. The expansion of the areas of co-presence of the two tick species could enhance the diffusion of the types for which both of them are competent.

## Conclusions

In the epidemiological systems involving *I. ricinus* as a vector, climatic changes have been shown to have several effects. First, the seasonal activity of ticks could undergo some changes [40,81]. Indeed, previous studies conducted on *I. ricinus* showed that this species could prolong its questing season, usually spanning from March to November, until January as a response of milder winters due to the temperature increase [3,81-84]. As a consequence, more abundant populations of ticks could survive the winter, thus a higher probability of tick bites, and in turn of disease transmission, could be expected [82]. Second, different life stages could become active and search for a host simultaneously [32,40]. Larvae and nymphs could parasitize the same host individual at the same time, favoring the trans-stadial transmission of pathogens by co-feeding and enhancing the efficiency of disease transmission [40,85]. In addition, our results suggest a potential significant increase of climatic niche of *I. ricinus* in the future years under both scenarios. Therefore disease outbreaks of pathogens transmitted by this tick species could emerge in previous non-endemic geographic areas. Further studies will implement and refine present data toward a better understanding of the risk represented by *I. ricinus* for human health.

## Competing interests

The authors declare that they have no competing interests.

## Authors’ contributions

DP, VM, SA SU, conceived and designed the study. DP, VM, SA drafted the manuscript. VM, SA analyzed the data. SG, SE, CG, CB, DO critically revised the manuscript. All authors read and approved the final manuscript.

## Supplementary Material

Additional file 1 List of bioclimatic variables used to develop the Species Distribution Models and variable percent contribution to construct the models. Click here for file

Additional file 2 **Increase of geographic distribution of *Ixodes ricinus*, predicted by species distribution modelling.** The values under Current, 2050 and 2080 (A2 and B2 scenarios) columns represent the areas in square km. The values in the Ratio column represent the increase of area as ratio between future and current conditions. Click here for file
