# Supplementary material for: Effects of global changes on the climatic niche of the tick Ixodes ricinus inferred by species distribution modelling
Source: Parasit Vectors. 2013 Sep 19;6:271. doi: 10.1186/1756-3305-6-271 (PMC3848450; doi:10.1186/1756-3305-6-271)
Supplement: Additional file 1 — List of bioclimatic variables used to develop the Species Distribution Models and variable percent contribution to construct the models. [file 1756-3305-6-271-S1.doc]

**Table S1**. List of bioclimatic variables used to develop the Species Distribution Models and variable percent contribution to construct the models.

| **Bioclimatic Variables** | **Variable Percent Contribution** | | | |
| --- | --- | --- | --- | --- |
|  | 2050 | | 2080 | |
|  | A2 | B2 | A2 | B2 |
| BIO1 = Annual mean temperature | 0.3 | 0.4 | 0.3 | 0.3 |
| BIO3 = Isothermality (BIO1/BIO7) * 100 | 0.2 | 0.3 | 0.3 | 0.4 |
| BIO6 = Min Temperature of Coldest Period | 39.3 | 39.4 | 39.7 | 38.8 |
| BIO8 = Mean Temperature of Wettest Quarter | 3.9 | 3.3 | 3.8 | 4.2 |
| BIO9 = Mean Temperature of Driest Quarter | 7.9 | 8.1 | 8.4 | 8.3 |
| BIO10 = Mean Temperature of Warmest Quarter | 1.4 | 1.3 | 1.4 | 1.2 |
| BIO11 = Mean Temperature of Coldest Quarter | 0.8 | 0.4 | 0.6 | 0.9 |
| BIO12 = Annual Precipitation | 10.7 | 10.1 | 10.2 | 9.4 |
| BIO13 = Precipitation of Wettest Period | 0.1 | 0.1 | 0.1 | 0.1 |
| BIO14 = Precipitation of Driest Period | 4.0 | 6.1 | 9.6 | 4.8 |
| BIO15 = Precipitation Seasonality (Coefficient of Variation) | 1.4 | 2.0 | 1.0 | 1.5 |
| BIO16 = Precipitation of Wettest Quarter | 0.1 | 0.1 | 0.1 | 0.1 |
| BIO17 = Precipitation of Driest Quarter | 26.7 | 24 | 21.4 | 25 |
| BIO18 = Precipitation of Warmest Quarter | 0.6 | 0.3 | 0.5 | 0.4 |
| BIO19 = Precipitation of Coldest Quarter | 2.7 | 4.1 | 2.8 | 4.3 |
