# Supplementary material for: Effects of global changes on the climatic niche of the tick Ixodes ricinus inferred by species distribution modelling
Source: Parasit Vectors. 2013 Sep 19;6:271. doi: 10.1186/1756-3305-6-271 (PMC3848450; doi:10.1186/1756-3305-6-271)
Supplement: Additional file 2 — Increase of geographic distribution of Ixodes ricinus, predicted by species distribution modelling. The values under Current, 2050 and 2080 (A2 and B2 scenarios) columns represent the areas in square km. The values in the Ratio column represent the increase of area as ratio between future and current conditions. [file 1756-3305-6-271-S2.doc]

**Table S2.** Increase of geographic distribution of *Ixodes ricinus*, predicted by species distribution modelling. The values under Current, 2050 and 2080 (A2 and B2 scenarios) columns represent the areas in square km. The values in the Ratio column represent the increase of area as ratio between future and current conditions.

|  |  |  | **A2** | **Ratio** | **B2** | **Ratio** |
| --- | --- | --- | --- | --- | --- | --- |
| **Current** | 3.1x10^6^ |  | **-** | **-** | **-** | **-** |
| **2050** | - |  | 6.9 x10^6^ | 2.2 | 7.4 x10^6^ | 2.4 |
| **2080** | - |  | 7.1 x10^6^ | 2.3 | 7.4 x10^6^ | 2.4 |
